# The Brazilian Society of Cardiology and Hypertension: It's Time for Action

**DOI:** 10.5935/abc.20180189

**Published:** 2018-09

**Authors:** Paulo César B. Veiga Jardim

**Affiliations:** 1 Departamento de Cardiologia da Faculdade de Medicina da Universidade Federal de Goiás, Goiânia, GO - Brazil; 2 Liga de Hipertensão Universidade Federal de Goiás, Goiânia, GO - Brazil

**Keywords:** Hypertension/physiopathology, Hypertension/mortality, Hypertension/drug therapy, Cardiovascular Diseases/prevention & control, Indicators of Morbidity and Mortality, Practice Guidelines as Topic, Antihypertensive Agents/administration & dosage, Decision/policies.

The year is 2018 and the level of scientific knowledge is unquestionable.

In medicine in general, and in cardiology, in particular, advances in diagnoses are impressive, just as the therapeutic arsenal is extraordinary, either in clinical or surgical, conventional or alternative treatment.

Consequently, longevity increases and the average age, even in our weakened Brazil, exceeds 75 years.^1^

Nevertheless, regarding the circulatory system diseases, the national picture is discouraging. With an older population, there are more degenerative diseases, and a higher proportional mortality from this cause.^1^

Among cardiovascular diseases, arterial hypertension and its consequences (stroke, coronary disease, heart failure, renal disease and peripheral vascular disease) undoubtedly achieve a record in terms of morbidity and mortality.^1^

Over the years, we have attended and participated in numerous initiatives of international and national scientific societies, with the purpose of establishing norms and behaviors for health professionals regarding care of hypertensive patients. The starting point was the Joint National Committee, organized in the United States from 1977 onwards, which for years determined the action standards that are considered more appropriate for treating hypertensive patients. Throughout the world, there has been the mobilization of scientific societies for the same purpose, always seeking to establish more correct and effective strategies.^2-4^

In Brazil, the initial Consensus and current Guidelines were first implemented in 1990 with a document of only 16 pages. It was held in the city of Campos do Jordão, and was an excellent work of the Brazilian societies of cardiology and nephrology that even led to the creation of the Brazilian Society of Arterial Hypertension.^5^

From then on, every 4 years there is an articulated mobilization of the scientific societies that work in this field (cardiology, nephrology, internal medicine, geriatrics, and other scientific societies in the health area - nutrition, physical education, nursing) and, in a collective effort, the documents are updated, new directions and strategies established and, occasionally, the guidelines for the treatment are changed.^6^

The philosophy of this mobilization was its broad dissemination at all levels of the health system, so that the instructions emanated from it could become current practice for the benefit of the patients. There were some advances, but even if, on the plane of intentions, at any moment, there was a regulation of the Ministry of Health itself to adopt the official documents of the scientific societies as rules for general action, the practical implementation of this expressed will has always followed the current political system, the temporary managers, and the country's own economic situation.

A great frustration!

Where were we and where are we?

Focusing our attention on Brazil, we find that, in general, from 1990 to the present, that is, in an interval of more than 25 years, little progress has been made.

It is a fact that the knowledge of the presence of hypertension by the population increased. We went from values lower than 50% of knowledge to numbers above 75%, and the merit was everyone's. The dissemination of the importance of the disease, and its identification, took place at all levels and, currently, few are unaware of the risks caused by hypertension. In this case, there was collective responsibility, and our scientific societies had an active participation in the dissemination process, either through its proselytism with health professionals, or through its action with the media and, in a significant way, its actions with the public power.^6^

If we make a simple and objective analysis of these numbers, we will see that, even with this advance, in every 100 hypertensive patients, 25 do not know their situation, and therefore do not even think about seeking treatment.

The percentage of individuals who are aware of their high blood pressure status, and who are undergoing treatment, has also increased, but in a less marked way when compared to those who are aware of the disease.

This number is around 65%, that is, in absolute numbers, among the 75 who know they have hypertension, approximately 50 individuals are being treated. So far, we have seen that in the group of hypertensives, out of 100, only half started treatment.^6^

Then we come to those in treatment, who have controlled pressure. This number is disappointing all over the world, but in Brazil it is even worse. If we analyze the available epidemiological data of treated individuals who present with controlled pressure, we have a percentage of control that reaches a maximum of 40%. The practical meaning of this number is that, in absolute values, only 25 individuals out of 100 hypertensive patients in general have their pressure controlled, that is, the old rule of halves, that we thought was outdated, is still valid.^6^

A resounding failure!

It is interesting to think back that we have advanced a lot!

If this state of affairs is perpetuated, morbidity and mortality from cardiovascular diseases will continue to be the highest, and may even increase, as the epidemic of overweight has also arrived in our country, with evident signs that it came to stay.

Still focusing on Brazil, where did we go wrong? How can we improve?

In daily practice, we still have difficulties in knowing about the presence of hypertension, but in this case, universal dissemination strategies have worked and only need to be reinforced and continued.

Regarding treatment, if on the one hand we have a very satisfactory therapeutic arsenal, on the other we still have many difficulties with its correct use.

There are several equally important aspects that should be actively addressed by our scientific societies. These include: a) medical training; b) continuing medical education; c) access to health services; (d) access to essential drugs; e) compliance to treatment.

The Brazilian Society of Cardiology has a serious responsibility, and should be more incisive, seeking to actively participate in the policy to control the creation and operation of medical schools and, at the same time, further increasing its participation in continuing medical education.

There should be concern about the critical review of our Guidelines. To whom they are intended, the extent of their content, how to implement them, and how to disseminate them throughout the country.

It is upsetting, and it is part of the real world, to see patients receiving inadequate treatment, either due to the type of drug used or to inadequate doses, while our guidelines and various international guidelines are dealing with ultra-advanced concepts that are not even possible to be performed in our practice.^6-10^

We have to rethink some issues.

The issue of access to services and key drugs should also be a matter of honor.

It is the duty of the organized society and of each individual citizen to participate in the pressure mechanisms so that public policies in the health area are effectively and continuously implemented. Thus, we can continue with so many projects that have already provided some progress and were completely lost due only to the political will of leaders who are not committed to society.

Perhaps, following this path, we will, in fact, contribute to a change in the history of cardiovascular diseases in our country.
